# The potential of ribonucleotide reductase M2 as a novel prognostic maker and therapeutic target to inhibit medulloblastoma proliferation, migration, and invasiveness

**DOI:** 10.1002/pdi3.81

**Published:** 2024-07-31

**Authors:** Xuanxuan Wu, Pan Gou, Chencheng Fang, Yudong Zhou, Lusheng Li, Xuan Zhai, Ping Liang

**Affiliations:** ^1^ Department of Neurosurgery, Children's Hospital of Chongqing Medical University National Clinical Research Center for Child Health and Disorders Ministry of Education Key Laboratory of Child Development and Disorders Chongqing Key Laboratory of Pediatrics Chongqing China

**Keywords:** invasiveness, medulloblastoma, migration, proliferation, RRM2

## Abstract

The crucial role of ribonucleotide reductase M2 (RRM2) enzyme in cancer occurrence and progression has been well‐established, but its specific function and significance in medulloblastoma (MB) remains largely unknown. First, we conducted a bioinformatics analysis of public genomic databases and observed highly expressed RRM2 in MB and an association of high RRM2 expression with adverse outcomes. In addition, by collecting clinical MB specimens for polymerase chain reaction (PCR), western blotting (WB), and immunohistochemistry (IHC), RRM2 was confirmed to be highly expressed in tumor tissues. Furthermore, immunohistochemical analysis linked adverse prognosis to high RRM2 expression. Moreover, knocking down RRM2 significantly inhibited MB cell proliferation, migration, and invasion in vitro. This report is the first to demonstrate the oncogenic role of RRM2 in MB, associated with adverse patient outcomes. Knocking down RRM2 contributes to weakened proliferating, migrating, and invading potentials of MB cells. RRM2 is expected to be a novel prognostic biomarker and therapeutic target for MB.

## Graphical abstract

The expression level of RRM2 is significantly higher in medulloblastoma tissues than in normal tissues. High expression predicts poor prognosis in patients with medulloblastoma of the transcriptomic profiles wingless (WNT) and non‐WNT/non‐sonic hedgehog (SHH) subtypes. Knocking down RRM2 in DAOY and ONS76 cells markedly inhibited cell proliferation, migration, and invasiveness.

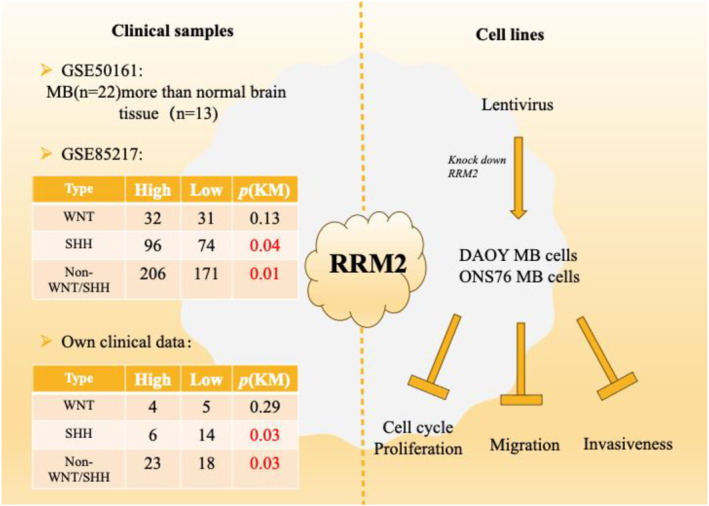

## 1 INTRODUCTION

Medulloblastoma (MB), a malignancy that originates from progenitor cells in and around the upper and lower rhombic lip,^1^ mainly occurs in the cerebellum and the brain stem.^2^, ^3^ Approximately, 75% of children with MB can survive into adulthood after receiving multidisciplinary treatment, which mainly includes surgery, radiotherapy, and chemotherapy. However, each treatment option may result in severe or delayed side effects, including but not limited to postsurgical neurocognitive disorders, muteness, endocrine disorders, infertility, and sterility, as well as an increased risk of secondary high‐grade meningiomas or gliomas. These complications can substantially degrade the quality of life for these children. Despite improved prognoses of MB children by comprehensive treatment, the recurrence rate remains as high as 20%–30%. Recurrences tend to occur more frequently within the initial 3 years following diagnosis, with approximately one‐third experiencing local recurrences, another one‐third developing metastases disseminated through the cerebrospinal fluid, and the final one‐third showing both types of recurrences.^4^, ^5^ Patients who experience recurrence after initial treatment have an extremely bleak outcome, with a median survival time of less than 6 months and a 2‐year survival rate of around 9%.

World Health Organization (WHO) recognizes four main histologic types of MB: classic, desmoplastic/nodular, extensive nodularity, and anaplastic and large cells. However, traditional pathological classifications can no longer effectively meet the increasingly complex clinical needs of today. The advancing comprehension of diseases necessitates a more refined and accurate classification system that incorporates diverse factors, including genetic mutations, molecular indicators, and distinct clinical features. There are four subgroups of MB based on genetic and transcriptomic profiles wingless (WNT), sonic hedgehog (SHH), Group 3, and Group 4. SHH and WNT subgroups are characterized by hyperactivated WNT and SHH pathways, while Group 3 and Group 4 MBs are defined by clustering algorithms without a clear understanding of key signaling pathways. Owing to the resemblance between Group 3 and Group 4 MB transcriptomes, coupled with the lack of identifiable driving mutations and signaling pathways, the WHO has recently categorized Group 3 and Group 4 MBs as non‐WNT/non‐SHH tumors.^6^ MB is highly malignant, and the mechanism at the molecular level needs further elucidation. Therefore, investigating the mechanisms underlying MB cell proliferation, migration, and invasiveness may shed valuable light on MB management.

Ribonucleotide reductase M2 (RRM2), predominantly located on chromosome 2, specifically at the p25–p24 region, is a crucial enzyme involved in DNA synthesis. Previous studies have demonstrated the significant role played by RRM2 in various types of cancer. In gastric cancer, RRM2 overexpression has been found to enhance invasiveness.^7^ In human glioblastoma, RRM2 inhibits cell apoptosis, promoting tumor progression.^8^ In gliomas, in vivo and in vitro experiments have demonstrated that RRM2 enhances cell growth and migration while also increasing sensitivity to radiotherapy. This enhancement in radiosensitivity is achieved by increasing the phosphorylation levels within the extracellular regulated protein kinases 1/2 and the serine/threonine kinase Akt signaling pathways.^9^ RRM2 is a promising therapeutic target for glioblastoma, and the use of triapine can effectively target RRM2, making glioblastoma cells more sensitive to radiation and inducing synthetic lethality when combined with checkpoint kinase 1 inhibition.^10^ Besides being a therapeutic target, RRM2 also serves as a diagnostic and therapeutic biomarker in liver cancer and breast cancer.^11^, ^12^ Moreover, it has been demonstrated that silencing RRM2 can increase the radiosensitivity of lung adenocarcinoma (LUAD) by synergistically boosting the signaling transduction of cyclic guanosine monophosphate–adenosine monophosphate synthase/stimulator of interferon genes.^13^ These results highlight the viability of focusing on RRM2 as a means to enhance the effectiveness of radiotherapy in individuals diagnosed with LUAD. Nunes et al. pinpointed RRM2 as a potential dependency factor, a finding that was bolstered by the observed growth inhibition following in vitro knockdown and the hastened tumor development in a neuroblastoma zebra fish model that coexpressed human RRM2 with MYCN.^14^ Despite the involvement of RRM2 in multiple cancer types, our current understanding of its expression profiling, mechanisms, and role in MB remains limited. Therefore, further research is warranted to investigate its contribution to MB and explore its therapeutic potential for this specific cancer.

Herein, we first demonstrated the highly expressed RRM2 in MB tissues versus normal brain tissues in the Gene Expression Omnibus (GEO) database. Kaplan–Meier (KM) curves additionally demonstrated a significant correlation between high expression of RRM2 and reduced overall survival (OS) in patients with MB. Furthermore, we confirmed through polymerase chain reaction (PCR), western blotting (WB), and immunohistochemistry (IHC) that RRM2 presented high expression in tumor tissues. Moreover, elevated RRM2 expression was indicative of a poor prognosis, as evidenced by IHC staining of tumor samples in conjunction with clinical prognostic data. KM curves and correlation analysis claimed that RRM2 could serve as a prognostic factor for MB. What's more, silencing RRM2 was demonstrated to be effective in preventing MB cells from proliferating, migrating, and invading, and inducing S‐phase arrest. Collectively, this paper provides comprehensive support for the significance of RRM2 as a biological prognostic marker in MB. These findings not only deepen our comprehension of the molecular processes behind MB development but also illuminate promising new avenues for drug therapy approaches targeting MB.

## 2 MATERIALS AND METHODS

### 2.1 Public data collection and analysis

GEO (URL: https://www.ncbi.nlm.nih.gov/geo/) is a globally accessible and comprehensive public repository providing high‐throughput microarrays, next‐generation sequencing, and functional genomic datasets, with data from a variety of pathological and normal specimens.^15^ For our analysis, we selected two key datasets: GSE50161, which contains genetic experimental microarrays from tumor tissues (*n* = 22) and normal counterparts (*n* = 13), and GSE85217, which includes genetic experimental microarrays and relevant clinical data from 763 patients. We utilized the online tool GEO2R (URL: https://www.ncbi.nlm.nih.gov/geo/geo2r/) to perform a comprehensive analysis of RRM2 differential expression patterns between MB and normal brain tissues using the original expression profile data obtained from the GSE50161 dataset. Pathway enrichment analysis of RRM2 was conducted using the Reactome database (URL: https://reactome.org/). To examine the correlation between RRM2 expression and patient survival outcomes, we integrated RRM2 expression profile information from the GSE85217 dataset with clinical data. Subsequently, we conducted KM survival analysis categorized by molecular subtypes, utilizing GraphPad Prism 9 for the analysis.

### 2.2 Participants and specimens

A total of 139 surgical specimens of MB were collected from pediatric patients who underwent surgical interventions at the Children's Hospital of Chongqing Medical University from 2012 to 2021. The obtained specimens were formalin‐immobilized and paraffin‐embedded. Out of these participants, 96 underwent molecular typing to further characterize their tumors. Follow‐up information was available for 70 patients out of the 139, enabling posttreatment evaluation and analysis. The collected tumors from the patients were categorized into distinct subgroups (WNT, SHH, or non‐WNT/non‐SHH) in accordance with the 2021 WHO Classification of Tumors of the Central Nervous System.^6^ Written informed consent was obtained from the guardians of all children, and the research process strictly adhered to the ethical principles outlined in the Helsinki Declaration.

### 2.3 Cells and cell culture

The cell lines DAOY SHH MB, ONS76 SHH MB, and SVGp12 astrocytes were obtained from the American Type Culture Collection (ATCC, USA). We maintained the DAOY, ONS76, and SVGp12 cells in Dulbecco's Modified Eagle Medium (DMEM, Gibco, USA) that was fortified with 10% (v/v) fetal bovine serum (FBS, Bio‐Channel, China). Cultivation of these cells occurred in an environment set at a constant temperature of 37°C and was humidified, containing 5% CO_2_ for optimal growth conditions.

### 2.4 RNA isolation and quantitative reverse transcription PCR (qRT‐PCR)

We isolated total RNA from the samples using the RNAiso reagent (Takara, Japan) and reverse‐transcribed it using the RNA PCR (AMV) kit (Takara, Japan). For PCR, we used primers that were designed for human genes, with glyceraldehyde‐3‐phosphate dehydrogenase as a control (see Table 1 for the sequence and product size of each primer pair). The experiment was run in triplicate under the following cycling conditions: 95°C for 5 min and 40 cycles of 95°C for 10 s, 60°C for 15 s, 72°C for 20 s, and 85°C for 5 s. The relative gene expression level was calculated using the 2^−ΔΔCt^ method.

**TABLE 1:** Primer information of real‐time qPCR.

Gene	Primer sequence(5′‐3′)
RRM2	Fwd 5′ TGGTCGACAAGGAGAACACG
	Rev 5′ CCAGGCATCAGTCCTCGTTT
GAPDH	Fwd 5′ CAACGGGAAACCCATCACCA
	Rev 5′ ACGCCAGTAGACTCCACGACAT

Abbreviation: GAPDH, glyceraldehyde‐3‐phosphate dehydrogenase.

### 2.5 WB

We collected MB cells and lysed them in ice‐cold Radio‐Immunoprecipitation Assay (RIPA) buffer (Beyotime Biotech, China) containing phenylmethanesulfonyl fluoride (PMSF, Beyotime), a protease inhibitor, and incubated them on ice for 20 min. After centrifuging the lysate at 13,000 rpm and 4°C for 20 min, the supernatant was collected to determine its protein concentration using the BCA protein assay (Beyotime). We loaded equal amounts of protein samples (20 μg/well) onto 10% sodium dodecyl sulfate–polyacrylamide gel electrophoresis (SDS‐PAGE) gels and separated them. The separated proteins were then transferred onto polyvinylidene difluoride (PVDF) membranes (Millipore, USA) at 4°C. We blocked the membranes with Phosphate‐Buffered Saline Tween (PBST)‐5% BSA and incubated them overnight at 4°C with primary antibodies (Proteintech, China). The membranes were then treated with corresponding secondary HRP‐conjugated antibodies (Proteintech) for 2 h at room temperature, and protein visualization and detection were performed using the FUSION FX.EDGE SPECTRA and ChemiDocXRS system, respectively.

### 2.6 IHC

After deparaffinization and rehydration through a graded ethanol series, antigen retrieval, and sealing, tissue slides were incubated with primary antibodies (Proteintech) overnight at 4°C. The slides were rinsed with phosphate‐buffered saline (PBS) and then incubated with the corresponding horse‐radish peroxidase (HRP)‐conjugated secondary antibody (Max Biotechnologies, China) for 30 min at 37°C. Following this, the slides were stained with 3, 3′‐diaminobenzidine (DAB, Max Biotechnologies, China) and counterstained with hematoxylin. MB specimens were subgrouped using IHC analyses as previously described, and immunohistochemical analysis was performed on 70 MB specimens. For each slide, a total of five random immunohistochemistry (IHC) images were captured using an Olympus BX53 microscope from Olympus, Japan. Image Fiji software was utilized to measure the sum of the positive site areas and the integrated optical density (IOD) sum in pixels. The RRM2 intensity was expressed as the mean of the IOD sum divided by the area sum of the five randomly selected photos for each slide. To ensure comparability, all photos were taken using the same settings. The optimal RRM2 intensity cutoff was determined to be 0.462, where samples with RRM2 intensity ≥0.462 were classified as having high RRM2 expression.

### 2.7 Plasmid and lentivirus preparation and infection

The pLKO.1 vector was used to introduce RRM2‐shRNA and GFP‐shRNA (see Table 2 for the target sequence). Lentiviruses were prepared with Lipofectamine 8000 (Beyotime Biotech, Shanghai, China), and shRNA plasmids were co‐transfected with three packaging plasmids (PAX2, VSVG) into HEK293FT cells for 48 h. After infection with lentivirus and polybrene (final concentration: 4 μg/mL) twice, MB cells were screened with puromycin (1.5 mg/mL) for follow‐up assays.

**TABLE 2:** Target sequence of shRNA.

Gene	Target sequence
RRM2	GCTCAAGAAACGAGGACTGAT
GAPDH	CCTCAACTACATGGTTTACAT

Abbreviation: GAPDH, glyceraldehyde‐3‐phosphate dehydrogenase.

### 2.8 Cell proliferation assay and colony forming assay

DAOY and sh‐DAOY, as well as ONS‐76 and sh‐ONS76 cells, were seeded into 96‐well plates at a density of 2000 and 1500 cells per well in 100 μL of culture medium, respectively. The IncuCyte real‐time video imaging system (Essen Instruments, Ann Arbor, MI, USA) was used to record cell growth curves. For the colony forming assay, cells were plated into 6‐well plates at a density of 1 × 10^3^ cells per well after 48 h of transfection. After 1 week, the colonies were fixed with 4% paraformaldehyde at room temperature for 30 min and stained with 0.5% crystal violet at room temperature for 30 min. Colony counting was performed using a light microscope.

### 2.9 Flow cytometry (FCM) analysis

Cells were preserved using 75% ethanol at a temperature of 4°C for a duration of 48 h, followed by staining with a propidium iodide (PI) solution that included RNase, at a temperature of 37°C. Subsequently, cell cycle analysis was conducted utilizing flow cytometry (BD LSRFortessa™, Becton, Dickinson Biosciences), adhering to the guidelines provided by the manufacturer.

### 2.10 Wound‐healing assays

Upon reaching complete confluence in the tumor cells cultured within 6‐well plates, a linear scratch was meticulously generated using a 200‐μL pipette tip. The cells were then cultured in FBS‐free DMEM for 48 h. Wound widths were observed microscopically and photographed at 0 and 48 h after the scratch was made.

### 2.11 Invasion transwell assays

Matrigel invasion assays were performed using 8‐μm‐pore‐size transwell cell culture chambers (24 wells; Corning, USA). The upper inserts, precoated with Matrigel (10 μL, Matrigel: DMEM = 1:3, v/v; BD, USA), were seeded with tumor cells (4 × 10^4^) that had been previously resuspended in serum‐free DMEM (200 μL). The lower chambers were filled with 10% FBS‐containing DMEM (500 μL). After 24 h, the invading cells were fixed and stained with crystal violet (CV), and non‐invading cells on the upper membrane were removed using cotton swabs. Invading cells were counted in five fields under an optical microscope (×200). The assays were conducted in triplicate or more.

### 2.12 Statistics and methods

All tests were performed in triplicate or more. The mean ± standard deviation (SD) was used for statistical description of the data. Statistical analysis was conducted using SPSS 20.0 and GraphPad Prism software to determine significant differences (*p*‐value <0.05) between the testing and control groups. The association between RRM2 and MB subgroups was assessed using statistical tests, including Student's *t*‐test and chi‐square analysis. To determine the statistical significance, a two‐tailed unpaired Student's *t*‐test or one‐way analysis of variance was performed. Patient survival analysis was conducted using KM curves and log–rank analysis.

## 3 RESULTS

### 3.1 Highly expressed RRM2 is correlated with adverse prognosis of MB in public database

**FIGURE 1:**
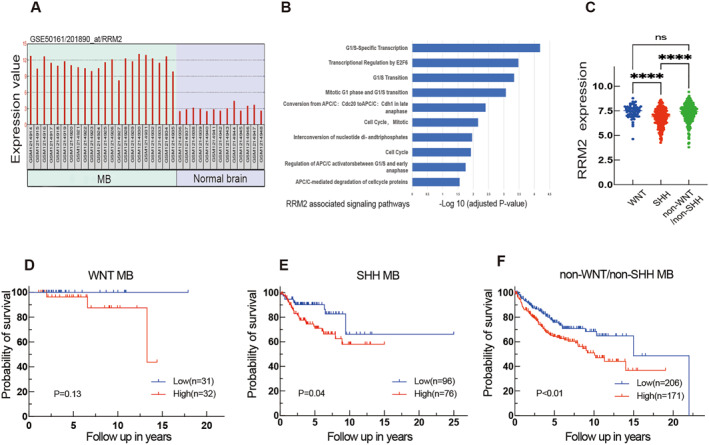
Bioinformatics analysis results. (A) Analysis of RRM2 mRNA levels in MB (*n* = 22) and normal tissues (*n* = 13). (B) Single gene enrichment analysis of RRM2. (C) Differences in RRM2 expression levels among different molecular subtypes of MB. (D, E, F) Analysis of the prognostic significance of RRM2 in different molecular subgroups of MB via the GSE85217 dataset, WNT(*n* = 63), SHH (*n* = 172), and non‐WNT/non‐SHH (*n* = 377). In addition to survival data, which were expressed as median, other data were given mean ± SD. Student's *t*‐test was performed to analyze significance; log‐rank (Mantel–Cox) test was carried out to analyze prognostic significance; ns *P* > 0.05, ****P < 0.0001.

In this study, we initially investigated RRM2 expression in the GEO GSE50161 dataset and observed significantly higher levels in MB compared to normal brain tissue (Figure 1A). Single gene pathway enrichment analysis using Reactome revealed a link between RRM2 and cell cycle regulation (Figure 1B). Additionally, analysis of the GEO GSE85217 dataset demonstrated significant differences in RRM2 expression between the WNT and SHH molecular subgroups, as well as between the SHH and non‐WNT/non‐SHH subgroups. However, no difference was observed between the WNT and non‐WNT/non‐SHH molecular subgroups (Figure 1C). Using the mean value as a benchmark to categorize RRM2 expression levels, subgroup‐specific KM analysis revealed that elevated RRM2 expression correlated with inferior OS in the non‐WNT/non‐SHH and SHH subgroups, but not in patients with WNT MB (Figure 1D–F). Therefore, high expression of RRM2 suggests a poorer prognosis in SHH and non‐WNT/non‐SHH MB cases.

### 3.2 RRM2 expression is higher in MB tissues than in paracancerous tissues (PT)

**FIGURE 2:**
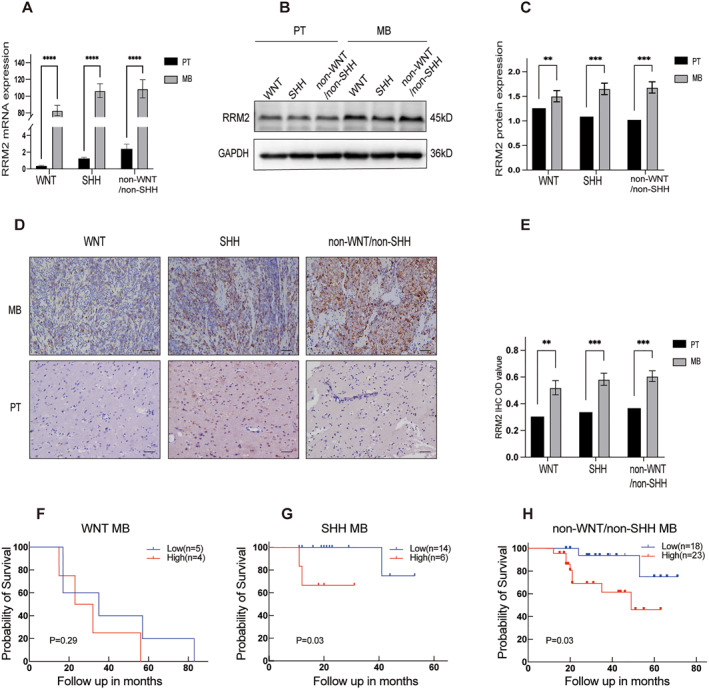
Clinical data analysis results. (A) PCR examination of RRM2 expression in different molecular subgroups of MB and PT. (B, C) WB examination of RRM2 expression in different molecular subgroups of MB and PT. (D, E) IHC staining of RRM2 expression in PT and MB samples, WNT(*n* = 9), SHH (*n* = 20), and non‐WNT/non‐SHH (*n* = 41), scale bar = 50 μm. (F, H) Analysis of the prognostic significance of RRM2 in different molecular subgroups of MB via our own clinical data. In addition to survival data, which were expressed as median, other data were given mean ± SD. Student's *t*‐test was performed to identify significance; log–rank (Mantel–Cox) test was carried out to analyze prognostic significance; **P < 0.01, ***P < 0.001, and ****P < 0.0001.

RNA was extracted from different molecular subtypes of MB and PT for PCR amplification. The PCR analysis revealed statistically significant higher RRM2 expression in tumor tissues compared to PT (Figure 2A). Additionally, proteins were isolated from various molecular subtypes of MB and PT, followed by the quantification of RRM2 protein levels through WB analysis. This analysis revealed a significant elevation in the expression of RRM2 protein in MB samples when contrasted with PT samples (Figure 2B,C). Furthermore, IHC staining images showed stronger RRM2 staining in the tumor compared to PT at the incisal edge (Figure 2D,E). These results provide evidence of high RRM2 in MB.

### 3.3 The correlation between RRM2 expression and clinical characteristics in MB patients

To further investigate the potential clinical significance and prognostic prediction value of high RRM2 expression, OS curves were plotted. In both the SHH and non‐WNT/non‐SHH subgroups, the high expression group exhibited a shorter survival time compared to the low expression group, indicating a poor prognosis (Figure 2F–H). Moreover, beyond OS, a correlation study was conducted to evaluate the association between RRM2 expression and diverse clinical characteristics. Complete information, including sex, age, WHO pathological type, and WHO molecular subgroup of MB, was collected and analyzed for MB patients. The results of the correlation analysis revealed a significant association between RRM2 expression and the molecular classification of MB (*p* = 0.047; Table 3).

**TABLE 3:** The relationship between RRM2 expression and clinic features in MB patients.

Characteristics	Total	RRM2 expression		*p* value
		Low	High	
	(*n* = 70)	(*n* = 36)	(*n* = 34)	
Gender				0.065
Male	39	21(53.8%)	18(46.2%)	
Female	31	15(48.4%)	16(51.6%)	
Age (years)				0.305
<3	14	9(64.3%)	5(35.7%)	
3–6	17	9(52.9%)	8(47.1%)	
6–14	39	18(46.2%)	21(53.8%)	
Histologic subtypes				0.787
Classic	47	23(48.9%)	24(51.1%)	
Desmoplastic/extensive nodularity	13	8(61.5%)	5(38.5%)	
MB with extensive nodularity	5	3(60.0%)	2(40.0%)	
Anaplastic and large cells	5	2(40.0%)	3(60.0%)	
Molecular subgroups				**0.047**
WNT	9	6(66.7%)	3(33.3%)	
SHH	20	14(70.0%)	6(30.0%)	
Non‐WNT/SHH	41	16(39.0%)	25(61.0%)	

### 3.4 Silencing RRM2 prevents MB cells from proliferating and induces S‐phase arrest

**FIGURE 3:**
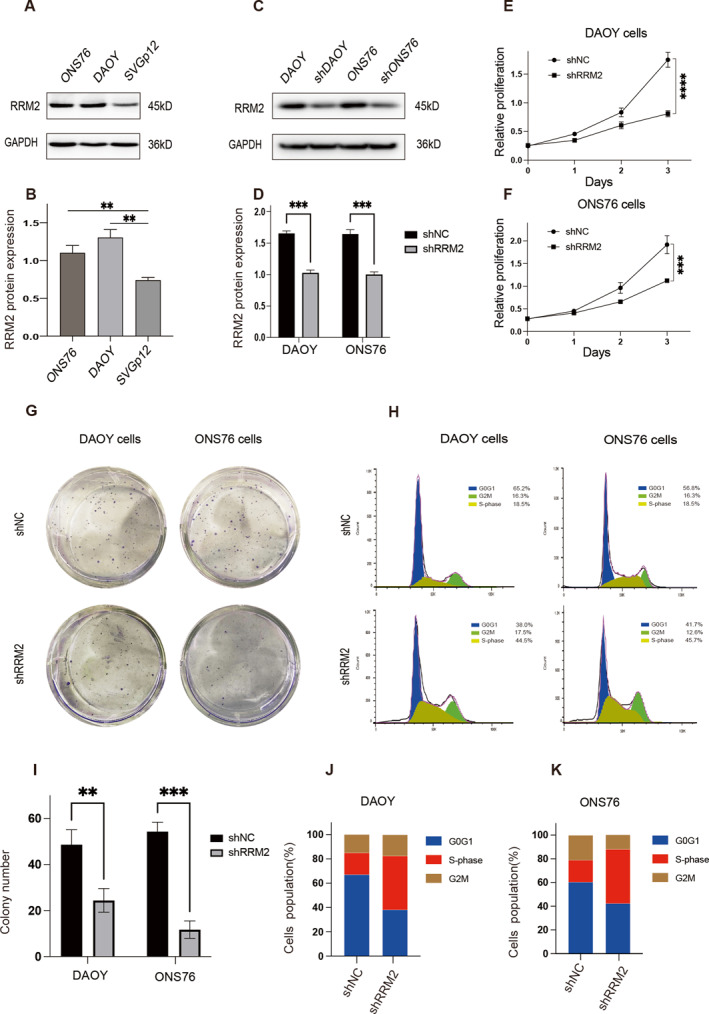
Cell proliferation assay results. (A, B) The protein levels of RRM2 were determined in DAOY, ONS76, and SVGp12. (C, D) The protein levels of RRM2 were determined in RRM2 knocked down MB DAOY and ONS76. (E, F) The proliferation ability of RRM2 knocked down MB cells was evaluated by IncuCyte real‐time video imaging system. (G, I) The colony ability of RRM2 knocked down MB cells was evaluated; scale bar, above: 50 mm. (H, J, and K) The growth curve and cell cycle was performed in DAOY and ONS76 after RRM2 knockdown. All data were given mean ± SD. Student's *t*‐test was performed to identify significance, **P < 0.01 and ***P < 0.001. NC is short for negative control.

To investigate RRM2 differential expression patterns in MB cells, WB analysis was performed on DAOY, ONS76, and SVGp12 cells (Figure 3A,B). Higher RRM2 expression was found in DAOY and ONS76 cells compared to SVGp12 cells. To evaluate the gene‐silencing efficiency of shRNAs specifically targeting RRM2, DAOY and ONS76 cells were transfected with these shRNAs, and RRM2 knockdown was confirmed using WB analysis (Figure 3C,D). Additionally, cell proliferation assays demonstrated that RRM2 knockdown effectively reduced the proliferative potential of DAOY and ONS76 cells (Figure 3E,F). Colony‐forming assays also showed a decreased colony count after RRM2 knockdown (Figure 3G,I). Furthermore, flow cytometry analysis revealed that RRM2 knockdown induced S‐phase arrest in MB cells (Figure 3H,J,K). These results indicate that RRM2 knockdown inhibits MB cell proliferation and induces S‐phase arrest.

### 3.5 Silencing RRM2 weakens the migrating and invading capacities of MB cells

**FIGURE 4:**
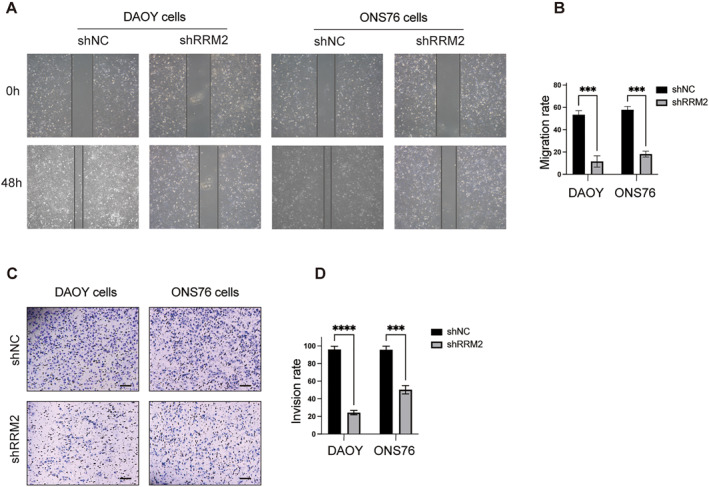
Cell migration and invasiveness assay results. (A, B) The migration abilities of RRM2 knocked down MB cells were evaluated by wound‐healing assay. (C, D) The invasiveness abilities of RRM2 knocked down MB cells were evaluated by transwell with matrix glue. All data were given mean ± SD. Student's *t*‐test was carried out to identify significance, ***P < 0.001, and ****P < 0.0001. NC is short for negative control.

To evaluate the impact of RRM2 silencing on cell migration and invasiveness, wound‐healing and transwell assays were performed. These assays were employed to examine the effects of inhibiting RRM2 on the ability of cells to migrate and invade. After RRM2 knockdown, a significant reduction in migrating potential was observed (Figure 4A,B). Similarly, the transwell assay demonstrated an inhibited invading capacity (Figure 4C,D). Statistical analysis of three independent experiments further confirmed significant reductions in both invasion and migration rates. These results provide evidence for the weakened migrating and invading potentials of MB cells following RRM2 silencing.

## 4 DISCUSSION

MB, the most commonly seen malignancy of the cerebellum in childhood, presents with an overall incidence rate of approximately 1.5 per million population in the United States of America.^16^ According to the WHO criteria, the disease is classified into WNT‐activated MB, SHH‐activated and TP53‐wildtype MB, SHH‐activated and TP53‐mutant MB, and non‐WNT/non‐SHH MB.^6^ WNT MB has the best prognosis and non‐WNT/non‐SHH MB with MYC amplification has the worst while the prognosis of MB patients with the SHH or non‐WNT/non‐SHH subgroup with MYC and CDK6 amplification is intermediate between these two, and that of SHH MB with TP53 mutations is extremely poor. Hence, studying the molecular‐level mechanism underlying non‐WNT/non‐SHH and SHH MB progression and prognosis is essential, as our findings could offer novel targets for MB treatment.

RRM2 is usually expressed at a high level in various cancers (e.g., colorectal, non‐small cell lung, breast, bladder, ovarian, pancreatic, head and neck, and lung carcinomas)^17^, ^18^, ^19^, ^20^ and is considered a prognostic marker for non‐small cell lung, cervical, and pancreatic cancers.^21^, ^22^ Additionally, suppressing RRM2 is reported to be effective in prolonging survival in glioma patients.^23^ RRM2 regulated antitumor immune responses, and knockdown of RRM2 enhances the antitumor efficiency of programmed cell death protein 1 blockade in renal cancer.^24^ Guo et al. previously identified RRM2 as a key gene in medulloblastoma through bioinformatics analysis, but they did not conduct any related experimental validation.^25^ Herein, we also found upregulated RRM2 in MB tissue through microarray analysis in GSE50161. Furthermore, an analysis of GSE85217 revealed a significant link between RRM2 expression and patient survival, with high levels of RRM2 expression correlating with poor outcomes in both SHH and non‐WNT/non‐SHH MB subgroups. Additionally, RRM2's involvement in cell cycle regulation was highlighted by single gene pathway enrichment analysis in the Reactome database. Therefore, RRM2 is anticipated to serve as a valuable biomarker for predicting the proliferation and prognosis of SHH and non‐WNT/non‐SHH MB.

In our study, we employed PCR, WB, and IHC techniques to validate the increased levels of RRM2 in tumor specimens obtained from our clinical cohort. By semi‐quantitative analysis of IHC staining in 70 samples, we observed a strong association between high RRM2 expression and unfavorable prognosis in MB patients, particularly those belonging to the SHH or non‐WNT/non‐SHH subgroups. Furthermore, analyzing clinical data from these 70 patients, we identified a correlation between RRM2 expression levels and specific molecular subtypes of MB. However, we did not observe significant correlations between RRM2 expression and patient gender, age, histopathological features, or tumor size. These findings further support the implication of RRM2 expression in the molecular classification of MB and emphasize its potential as a promising and novel prognostic marker for SHH and non‐WNT/non‐SHH MB subgroups.

Actually, high RRM2 expression stimulated the proliferating, migrating, and invading potentials of multiple solid tumors cells. RRM2, an upstream regulator of the ovarian cancer cell cycle,^26^ has also been shown to promote gastric cancer cell invasiveness and migration.^27^ Other studies have revealed reduced invasiveness capacity of estrogen receptor‐negative breast cancer cells,^28^ altered colorectal cancer cell proliferation under a serum‐depleted condition,^17^ and even suppressed cellular growth in head and neck squamous cell and non‐small cell lung carcinomas after inhibiting RRM2.^29^ In our vitro studies, we discovered that silencing RRM2 led to a significant inhibition of MB cell proliferation, suggesting that RRM2 might act as a regulator of the cell cycle, particularly during the S‐phase. To further investigate the regulatory role of RRM2 in the cell cycles of DAOY and ONS‐76 MB cell lines, we conducted cell cycle analyses using FCM. The results demonstrated that following the knockdown of RRM2, there was a noticeable arrest of these cells in the S‐phase, indicating a critical function of RRM2 in facilitating cell cycle progression within MB cells. Moreover, knocking down RRM2 was effective in suppressing the migrating and invading potentials of DAOY and ONS‐76, as evidenced by transwell and wound‐healing assays. To sum up, RRM2 is the upstream regulator of human MB cell proliferation, migration, invasiveness, and cell cycle.

## 5 CONCLUSION

This study provides evidence of elevated RRM2 levels in human MB and establishes a link between high RRM2 expression and unfavorable prognosis in MB patients belonging to the SHH or non‐WNT/non‐SHH subgroups. Additionally, RRM2 is identified as an upstream regulator of MB cell proliferation, migration, invasiveness, and cell cycle. Overall, these findings highlight the cocarcinogenic role of RRM2 in human MB and suggest its potential as a novel prognostic marker and therapeutic target for this disease.

## AUTHOR CONTRIBUTIONS

All the authors have strong, direct, and effective contributions to this research and agree to publish it. Xuanxuan Wu.: conceptualization, methodology, investigation, writing‐original draft, and formal analysis; Pan Gou: Resources, investigation, and data curation; Chencheng Fang: methodology, resources, and visualization; Yudong Zhou: visualization, investigation, writing‐review & editing; Lusheng LI and Xuan Zhai: resources, writing‐review & editing, supervision, and funding acquisition; Ping Liang: conceptualization and funding acquisition.

## CONFLICT OF INTEREST STATEMENT

The authors declare no competing interests.

## ETHICS STATEMENT

The clinical information and specimens used in this article were approved by the Ethics Committee of Children's Hospital of Chongqing Medical University [2023(581)]. Written informed consent was obtained from the guardians of the patients.

## Data Availability

The data used to support the finding is available in the GEO dataset (GSE50161 and GSE85217). Raw clinical data can be available upon reasonable request to authors.
